# Synergistic AHR Binding Pathway with EMT Effects on Serous Ovarian Tumors Recognized by Multidisciplinary Integrated Analysis

**DOI:** 10.3390/biomedicines9080866

**Published:** 2021-07-22

**Authors:** Kuo-Min Su, Hong-Wei Gao, Chia-Ming Chang, Kai-Hsi Lu, Mu-Hsien Yu, Yi-Hsin Lin, Li-Chun Liu, Chia-Ching Chang, Yao-Feng Li, Cheng-Chang Chang

**Affiliations:** 1 Graduate Institute of Medical Sciences, National Defense Medical Center, Taipei 114, Taiwan; aeolusfield@hotmail.com (K.-M.S.); hsienhui@ms15.hinet.net (M.-H.Y.); 2 Department of Obstetrics and Gynecology, Tri-Service General Hospital, National Defense Medical Center, Taipei 114, Taiwan; m860371@gmail.com (Y.-H.L.); lvita.tw@gmail.com (L.-C.L.); t791212@gmail.com (C.-C.C.); 3 Department of Pathology, Tri-Service General Hospital, National Defense Medical Center, Taipei 114, Taiwan; doc31796@gmail.com; 4 School of Medicine, National Yang Ming Chiao Tung University, Taipei 112, Taiwan; cm_chang@vghtpe.gov.tw; 5 Department of Obstetrics and Gynecology, Taipei Veterans General Hospital, Taipei 112, Taiwan; 6 Department of Medical Research and Education, Cheng-Hsin General Hospital, Taipei 112, Taiwan; lionel.lu@gmail.com; 7 Division of Obstetrics and Gynecology, Tri-Service General Hospital Songshan Branch, National Defense Medical Center, Taipei 105, Taiwan

**Keywords:** gene ontology, epithelial ovarian cancers, borderline ovarian tumors, differentially expressed genes, aryl hydrocarbon receptor, epithelial–mesenchymal transition, integrative analysis

## Abstract

Epithelial ovarian cancers (EOCs) are fatal and obstinate among gynecological malignancies in advanced stage or relapsed status, with serous carcinomas accounting for the vast majority. Unlike EOCs, borderline ovarian tumors (BOTs), including serous BOTs, maintain a semimalignant appearance. Using gene ontology (GO)-based integrative analysis, we analyzed gene set databases of serous BOTs and serous ovarian carcinomas for dysregulated GO terms and pathways and identified multiple differentially expressed genes (DEGs) in various aspects. The *SRC* (SRC proto-oncogene, non-receptor tyrosine kinase) gene and dysfunctional aryl hydrocarbon receptor (AHR) binding pathway consistently influenced progression-free survival and overall survival, and immunohistochemical staining revealed elevated expression of related biomarkers (SRC, ARNT, and TBP) in serous BOT and ovarian carcinoma samples. Epithelial–mesenchymal transition (EMT) is important during tumorigenesis, and we confirmed the *SNAI2* (Snail family transcriptional repressor 2, *SLUG*) gene showing significantly high performance by immunohistochemistry. During serous ovarian tumor formation, activated AHR in the cytoplasm could cooperate with SRC, enter cell nuclei, bind to AHR nuclear translocator (ARNT) together with TATA-Box Binding Protein (TBP), and act on DNA to initiate AHR-responsive genes to cause tumor or cancer initiation. Additionally, SNAI2 in the tumor microenvironment can facilitate EMT accompanied by tumorigenesis. Although it has not been possible to classify serous BOTs and serous ovarian carcinomas as the same EOC subtype, the key determinants of relevant DEGs (*SRC, ARNT, TBP*, and *SNAI2*) found here had a crucial role in the pathogenetic mechanism of both tumor types, implying gradual evolutionary tendencies from serous BOTs to ovarian carcinomas. In the future, targeted therapy could focus on these revealed targets together with precise detection to improve therapeutic effects and patient survival rates.

## 1. Introduction

Ovarian tumors occupy a certain place among gynecological diseases and most cases are benign in clinical and pathological features such as follicular cysts, corpus luteum cysts, serous or mucinous cystadenomas, endometriomas, and teratomas [1,2]. Comparatively, ovarian cancer is the most lethal gynecological malignancy worldwide although the proportion is relatively low [3]. Epithelial ovarian cancers (EOCs) are the leading cause of death among patients with gynecologic cancers accounting for the vast majority of all ovarian cancers [4,5]; furthermore, serous carcinoma (SC) accounts for the most common of EOCs, with a poor prognosis and a five-year survival rate of only 25% with metastases [6,7]. SC is less likely to be found in the early stages (International Federation of Gynecology and Obstetrics (FIGO) stages I and II), which have higher survival rates because they are easier to treat, whereas patients at advanced stages (FIGO stages III and IV) have poor prognosis and high recurrence rates even after complete debulking surgery combined with chemotherapy (carboplatin and paclitaxel) due to resistance to chemotherapy [6,8,9].

Borderline ovarian tumors (BOTs), a specific subtype of EOCs, consist of disparate groups of neoplasms based on histopathological features, molecular characteristics, and clinical behaviors and BOTs can generally be classified into serous, mucinous, and other subtypes according to clinical and histopathological features [10,11]. Besides, BOTs account for approximately 10–15% of EOCs and usually occur in younger women, resulting in an excellent prognosis [12]. Compared with ovarian cancer patients, who almost always require chemotherapy after a debulking operation, patients with BOTs usually have better prognoses after adequate surgery with an extremely low probability of recurrence or metastasis [13,14]. Serous BOTs, comprising approximately 65% of BOTs, occur mostly in North America, the Middle East, and most of Europe [15]. To date, surgery is still the ideal method to treat BOTs, while adjuvant chemotherapy and radiotherapy are not usually considered as standard therapies [14,16]. Recent studies have inferred several assumptions, including the incessant ovulation, gonadotropin, hormonal, and inflammation hypotheses, to explain the tumorigenesis of serous BOTs [17,18,19]. Serous BOTs are characterized by mutations in the *KRAS*, *BRAF*, and *ERBB2* genes and overexpression of the *p53* and *Claudin-1* genes; furthermore, the mitogen-activated protein kinase (MAPK)/extracellular signal-regulated kinase (ERK) pathway, PI3K/AKT/mTOR pathway, Hedgehog pathway, and angiogenesis pathway are frequently activated in serous BOTs [13,14,20,21,22,23,24,25].

As a complex disease, several genetic and environmental factors contribute to SC development with a complicated carcinogenesis pathway, and the carcinogenesis of SC evolves through several aberrant functions, which fluctuate with disease progression based on findings through the widely utilized FIGO system [13,26,27,28,29,30,31,32]. It is widely known that most serous ovarian carcinomas are associated with *TP53* mutations [30,33,34,35,36]; about half of them have undergone abnormal DNA repair processes through homologous recombination due to epigenetic or genetic alterations of *BRCA1*, *BRCA2*, or other DNA repair molecules [37,38]; and some show gene mutations, such as in BRAF and KRAS [20]. In addition to debulking surgery and subsequent adjuvant chemotherapy, targeted therapy and systemic immunotherapy can also be utilized to enhance the therapeutic effects. Poly-adenosine diphosphate (ADP) ribose polymerase inhibitors (PARPis), the first approved cancer drugs, were widely used targeted therapies for BRCA1/2-mutated breast and ovarian cancers, and they specifically target DNA damage and repair responses, especially for patients with homologous recombination deficiencies, resulting in increased survival [39,40,41]. However, resistance to PARPi has recently become an emerging issue and breast-related cancer antigens (BRCA) and homologous recombination deficiency (HRD) status can be considered novel predictive biomarkers of response [42,43,44,45]. Therefore, identifying potential crucial biomarkers for monitoring drug resistance and formulating new drug combination strategies are efficacious methods to resolve PARPi resistance along with precision medicine [46,47].

Furthermore, it is well known that serous ovarian carcinomas have a poor clinical prognosis because they are usually diagnosed too late, while advanced stages usually result in frequent emergence of chemoresistance [4,5,8,48,49]. Recent growing evidence suggests that epithelial–mesenchymal transition (EMT) may contribute to tumor invasion and metastasis and promote chemotherapeutic resistance, especially to cisplatin, by converting the motionless epithelial cells into mobile mesenchymal cells, escaping cell adhesion, and altering the cellular extracellular matrix [50,51,52]. EMT is a reversible process in which many crucial components, such as E-cadherin, EpCAM, vimentin, fibronectin, neural cadherin, matrix metalloproteinases, various integrins, and different cytokeratins, are regulated by a complex functional network of transcription factors, including the zinc-finger E-box-binding homeobox factors (Zeb1 and Zeb2), Snail (SNAI1), Slug (SNAI2), and the basic helix–loop–helix factors (Twist1 and Twist2) [53,54,55]. Loss of breast cancer type 1 susceptibility protein (BRCA1), a tumor suppressor that plays a role in mending double-stranded DNA breaks, is also associated with EMT and tumor initiation [50,56]. The expression of EMT signaling pathways has been correlated with poor prognosis in various epithelial cancers, including breast, pancreas, prostate, and ovarian cancer, and the role of EMT in ovarian cancer progression and therapy resistance is highlighted in current studies [57]; however, the role of EMT plasticity in serous ovarian tumors has not been comprehensively investigated.

As mentioned above, although both are named the “serous” subtype in terms of classification, serous BOTs and serous ovarian carcinomas still have decisive differences in genetic mechanisms, pathological characteristics, and clinical manifestations [13,32]. Various functions can be investigated using differentially expressed genes (DEGs) detected by microarrays. In contrast to DEGs, we established a gene set regularity (GSR) model, which reconstructed the functionomes, that is, the GSR indices of the global functions, and then investigated the dysregulated functions and dysfunctional pathways involved in the complex disease. Constructing a functionome can provide information about the dysregulated functionomes accompanied with dysfunctional pathways of complicated illness and we had conducted several gene set-based analyses by integrating microarray gene expression profiles downloaded from publicly available databases, which revealed that comprehensive methods based on functionome defined by gene ontology (GO) are useful for successfully conducting significant research on BOTs and ovarian carcinomas of different stages and subtypes [58,59,60,61,62,63,64]. Previously, individual studies have focused on gene set analysis of serous ovarian carcinomas and serous BOTs to uncover pathogenic mechanisms during tumorigenesis. However, there is no integrated analysis to compare and discover the genomic functionome of serous BOTs and serous ovarian carcinomas. Therefore, we first utilized GO-based integrative approaches to explore expression profile datasets of serous ovarian tumors, including serous BOTs and all stages of serous ovarian carcinomas, to identify common and meaningful dysregulated functions and dysfunctional pathways between these two groups. We then selected the featured DEGs by checking the significant biomarkers related to EMT with cross comparison. In this experiment, we aimed to excavate newly discovered pathogenetic mechanisms based on previous studies that differ from previous theories and hoped to take advantage of these new findings applied in medical detection with targeted therapy and effective avoidance of recurrence for better prognosis of serous ovarian tumors and patient survival.

## 2. Materials and Methods

### 2.1. Workflow for the Integrative Analytic Model

**Figure 1:**
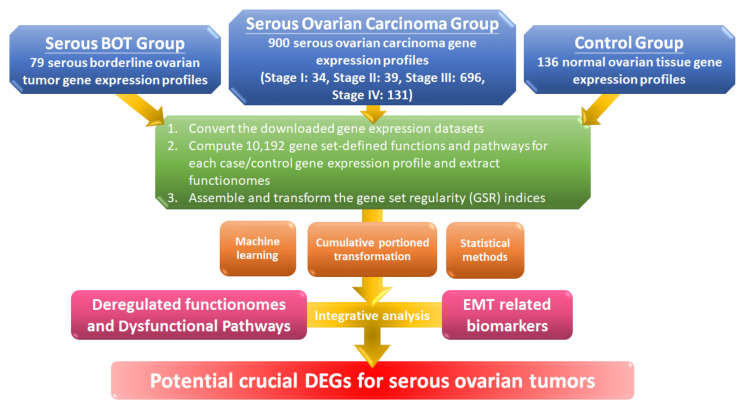
Workflow of this study. The DNA microarray gene expression profiles of 79 serous borderline tumor (BOT) samples, 900 serous ovarian carcinoma specimens including all stages, and 136 normal ovarian controls were downloaded from publicly available databases with gene set regularity (GSR) indices calculated by the Gene Ontology (GO) gene set. Functionomes consisting of 10,192 GO gene sets established from the polygenic models and cumulative portion transformations with machine learning and statistical methods were utilized to identify the functionome-based patterns to investigate dysregulated GO terms, dysfunctional pathways, and biomarkers of epithelial–mesenchymal transition (EMT) together with integrative analysis and to discover potential crucial differentially expressed genes (DEGs).

The workflow for this study is shown in the flowchart in Figure 1, and detailed information is explained below. First, we converted the gene expression profile of the extracted gene elements downloaded from the Gene Expression Omnibus (GEO) database with selection criteria for serous ovarian tumors and normal controls to ordered data and then transformed them into 10,192 quantified GO terms according to the sequential expression from the gene elements in each gene set. This process produced functionomes consisting of 10,192 GSR indices, which defined the relatively comprehensive biological and molecular functions to explore serous ovarian tumors, including serous BOTs and all stages of serous ovarian carcinomas. Next, we individually calculated the quantified functions and functional regularity patterns among serous ovarian tumors and 136 normal ovarian controls with GSR indices and established the GSR model for the functionome pattern. Then, we investigated the whole informativeness of genomic functionomes consisting of the GSR indices and constructed a functionome-based training model of classification and prediction using the support vector machine (SVM), a set of supervised mathematical commands from machine learning. The variation in the GSR indices between each serious ovarian tumor group and normal control group revealed that the biomolecular functions among serous ovarian tumors were significantly extensively dysregulated in contrast to the normal control group. Finally, we conducted whole-genome integrative analysis to identify meaningful dysfunctional pathways together with significant biomarkers of EMT involved in the progression of serous ovarian carcinomas to determine crucial DEGs that may be essential parts of the pathogenetic mechanisms for serous ovarian tumors by elucidating dysregulated functionomes using microarray analysis of gene expression profiles. The key biological functions and genes involved in the pathogenesis of serous BOTs and all stages of serous ovarian carcinomas were determined by identifying genome-wide and GO-defined functions and DEGs.

### 2.2. Microarray Dataset Collections and the Selection Criteria

The selection criteria for the microarray gene expression datasets from the GEO database were as follows: (1) samples of normal controls and serous ovarian tumor samples, including serous BOTs and serous ovarian carcinomas, should all originate from ovarian tissues of homo sapiens; (2) datasets should offer sufficient information about the diagnosis and clinical histopathological subtypes of serous ovarian tumors and normal controls should consist of tissues or cell cultures from normal ovarian surface epithelium (NOSE) judged by histology; and (3) any extracted sample that did not meet the above-mentioned conditions was discarded and any gene expression profile in a dataset was abandoned if it contained missing data.

### 2.3. Computing the GSR Indices and Rebuilding the Functionomes

GSR indices were calculated and extracted from the gene expression datasets by modifying the differential rank retention (DIRAC) algorithm [65] and used to measure sequential changes among the gene elements in the gene set datasets of the gene expression profiles of serous BOTs, all stages of serous ovarian carcinomas, and the most common gene expression ordering from the normal control samples. The details and calculation process of the GSR model were described in our previous studied papers [58,59,60,61,62,63,64]. The microarray-based gene expression profiles from serous ovarian tumors and normal ovarian samples obtained from the GEO database were produced using the corresponding gene expression levels constructed according to the genetic elements in the GO-based functionome, which were then con-verted into ordered data based on each expression level. By definition, the GSR index refers to the ratio of the gene expression sequence in a gene set between the case group and the most common gene expression sequence from the normal ovarian tissue samples, ranging from 0 to 1, where 0 represents the most dysregulated state of a gene set with oppositely ordered gene set regularities between the serous ovarian tumors and the most common gene expression orderings in the normal controls, whereas 1 indicates that the genomic regularities in a gene set remain the same between the groups of serous ovarian tumors and the normal ovarian group. All GSR indices were measured using the R programming language. A functionome was defined as the complete gene set of biological functions, and we annotated and defined the human functionome using the 10,192 GO gene set-defined functions because the definitions for comprehensive biological functions are not yet available. Therefore, the functionomes used in this study were defined as a combination of 10,192 GSR indices for all samples.

### 2.4. Statistical Analysis

The Mann–Whitney U-test was used to test the differences in serous BOTs, all stages of serous ovarian carcinomas, and controls, and then corrected by multiple hypotheses using the false discovery rate (Benjamini–Hochberg procedure) [66]. The *p*-value was set at *p* < 0.05.

### 2.5. Classification and Prediction by Machine Learning with Set Analysis

An R package with the function “kvsm” provided by the “kernlab” (version 0.9–27; Comprehensive R Archive Network) and kernel-based machine learning methods were used to classify and predict patterns of GSR indices. K-fold cross-validation was used to measure the accuracy of classification and prediction of SVM. The results of ten repetitive predictions were used to evaluate the performance of the binary classification. The R package “pROC” was used to calculate the area under the curve (AUC) [67]. The R package “data.table” (version 1.12.8; Comprehensive R Archive Network) was used to display all possible logical relationships among the dysregulated gene sets of serous ovarian tumors clearly and sequentially in the tables.

### 2.6. Verification of Clinical Samples Using Immunohistochemical (IHC) Staining Method

Fifty clinical samples of serous ovarian tumors were collected (serous BOTs, n = 9; serous ovarian carcinomas, n = 41, including 8 stage I, 2 stage II, 23 stage III, and 8 stage IV cases). All serous ovarian tumor tissues were collected from female patients undergoing surgical treatments after signing an informed consent agreement. All patients were diagnosed and treated according to the standard therapeutic guidelines, and all tissues of patients were kept in the biobank at Tri-Service General Hospital, National Defense Medical Center, Taipei, Taiwan. The Institutional Review Board of the General Hospital of the National Defense Medical Center approved the study (2-107-05-043, approved on October 26, 2018, and 2-108-05-091, approved on 20 May 2019). Informed consent was obtained from all patients and control subjects. All clinical tissue samples were confirmed via quantitative histopathological inspections and diagnosed by professional pathologists, and IHC staining results were scored as follows: the intensity (I) was multiplied by the percentage of positive cells (P) of all biomarkers utilized in this study (the formula is shown as IHC score [Q] = I × P; maximum = 300) [68,69].

## 3. Results

### 3.1. Microarrays of Sample Groups for Gene Expression Profiles and Definition for Gene Set Analysis

We performed a comprehensive bioinformatics method based on GO to explore and analyze all relational disordered functions of serous ovarian tumors, including serous BOTs and all stages of serous ovarian carcinomas [70]. The gene expression profiles of DNA microarray of serous ovarian tumors and normal controls were downloaded from the GEO repository at the National Center for Biotechnology Information (NCBI) archives. The whole-sample data were obtained from 30 datasets containing eight heterogeneous DNA microarray platforms without any missing data. There were 79 serous BOT samples and 900 serous ovarian carcinoma samples based on histopathological classification, including 34 stage I, 39 stage II, 696 stage III, and 131 stage IV cases among the 900 serous ovarian carcinoma samples according to the FIGO staging system (Table 1). In addition, 136 normal ovarian samples were collected as a control group for comparison. Table S1 provides detailed information about all obtained samples and controls. In total, 10,192 GO-based definitions for annotating all gene set-defined functionomes were also downloaded from the Molecular Signatures Database (MSigDB), the version “c5.all.v7.1.symbols.gmt” [71].

### 3.2. Histograms of GSR Indices of Functionomes among Each Group with Diverse Differences

**Figure 2:**
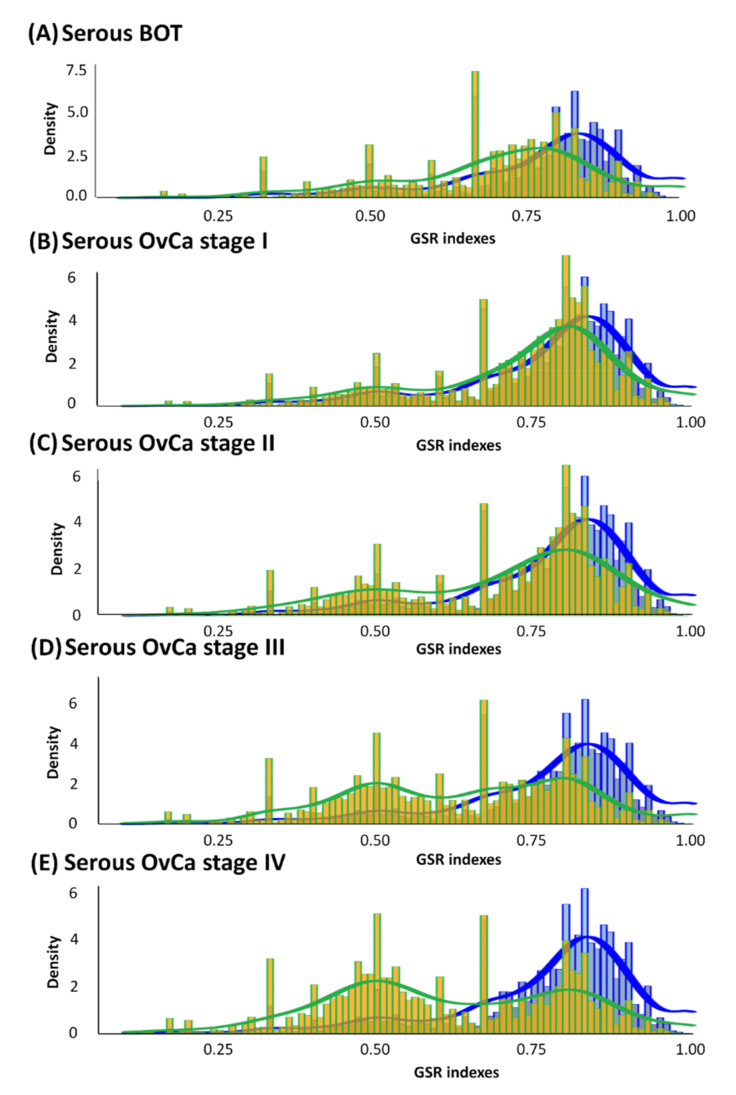
Histograms of global GSR indices of functionomes for serous BOTs, all stages of serous ovarian carcinomas (yellow green), and control groups (blue). Different distributions of the functionomes among five case sample groups and control groups are shown with statistical significance (*p* < 0.05). The normal control group (blue, right) was used as the control and is the same in all panels. Peaks in distribution were observed (yellow green), indicating dysregulated biomolecular functionomes of serous BOTs and all stages of serous ovarian carcinomas. (**A**–**E**) Corrected GSR indices of serous BOTs: 0.7036 (**A**); FIGO stage I: 0.7230 (**B**); FIGO stage II: 0.6976 (**C**); FIGO stage III: 0.6355 (**D**); and FIGO stage IV: 0.6147 (**E**).

According to the divergence in ranking within a gene set between the case and control groups characterized by GO terms, GSR indices were individually calculated by quantifying alterations in the ranking of gene expression in a gene set or functionome. As displayed in Figure 2, all averages of GSR indices for the functions of serous ovarian tumors were computed and then rectified by the mean values of the control group. Divergences in GSR indices between serous ovarian tumor and normal control groups were statistically significant (*p* < 0.05). We found that in the serous ovarian carcinoma group, as the FIGO stage progressed from early stages (I and II, yellow-green grids in Figure 2B,C, respectively) to advanced stages (III and IV, yellow-green grids in Figure 2D,E, respectively), the differences from corresponding normal controls (blue grids in Figure 2) became increasingly distinct. Furthermore, differences in mutations among serous BOTs (yellow-green grids in Figure 2A) and normal controls seemed to be more irregular than those at the early stages (FIGO stage I and II) but less aberrant than at the late stages (FIGO stage III and IV) of serous ovarian carcinomas. The modulation of dysregulated functionomes was quantified using the means of the total GSR indices of each functionome of serous ovarian tumors and control groups with adjustments compared to the control group. The average corrected GSR indices for serous BOTs and serous ovarian carcinomas from stage I to IV were 0.7036, 0.7230, 0.6976, 0.6355, and 0.6147, respectively.

### 3.3. Regularity Patterns of Functionomes Classified and Predicted by Supervised Machine Learning with High Sensitivity, Specificity, and Accuracy

As displayed in Figure 2, the regularity patterns of functionomes among the five serous ovarian tumor groups were compared with those of the normal control group, and the functional regularity patterns of the five case groups (serous BOTs and all four stages of serous ovarian carcinomas) showed significant divergence. We then identified, classified, and predicted different functions of various gene sets defined by GO using SVM, a powerful technological algorithm for supervised machine learning. The accumulated data of assessed performances were operated with ten consecutive binary classifications and checked with forecasting approaches by five-fold cross-validation; all the calculated results with high sensitivity, specificity, and accuracy are listed in Table S2. The sensitivity, specificity, and accuracy of binary classification for gene set databases among serous ovarian tumor and control groups were approximately 95.13–100.00%, 99.85–100.00%, and 98.97–100.00%, respectively. The AUCs for the performance of each case group ranged from 0.9771 to 1.0000. The evaluated performances by binary classifications between serous ovarian carcinoma, FIGO stage IV, and normal control groups had the best overall effects. The results revealed that the quantified functional regularity patterns with the GSR indices transformed from the DNA microarray gene expression profiles could offer sufficient and credible information to the SVM for accurate identification and classification. These results also indicated that all the functional regularity patterns of serous ovarian tumors were demarcated and suitable for integrated genetic and molecular classifications interpreted in this study.

### 3.4. The Most Dysregulated and Common GO Terms among Serous Ovarian Tumors

We used the cluster weight index (CWI) with SVM to uncover 655, 662, 643, 828, and 841 GO terms among serous BOTs and serous ovarian carcinomas at FIGO stages I–IV, respectively. CWI, a calculated exponent based on the *p*-values with statistical significance, is defined as the weighted ratio of the single weight of each clustered GO term divided by the total weights of the whole clusters, and it is used to measure the representative weight and express the mutual correlation for every cluster in the GO trees. All identified GO terms were meaningful and could represent dysregulated functionomes in each group of serous ovarian tumors. We used the calculated CWI to quantify and judge the value of each dysregulated GO cluster among the pathogenetic mechanisms of serous ovarian tumors. We ranked the 50 most dysregulated GO terms judged by CWI for serous ovarian tumors, as shown in Table 2. The first dysregulated GO terms for each group were “regulation of immune system process (GO:0002682)” for serous BOT; “transporter activity (GO:0005215)” for serous ovarian carcinoma, FIGO stage I; “small molecule metabolic process (GO:0044281)” for serous ovarian carcinoma, FIGO stage II; “regulation of immune system process (GO:0002682)” for serous ovarian carcinoma, FIGO stage III; and “small molecule metabolic process (GO:0044281)” for serous ovarian carcinoma, FIGO stage IV. Details on dysregulated GO terms for all disease groups of serous ovarian tumors are listed in Table S3. We then selected and reorganized the top 25 from the 50 most dysregulated GO terms among the five groups by comprehensively comparing weighted CWIs with their original rankings in each group, as listed in Table S4. Next, we summarized the 25 most common dysregulated GO terms among the five disease groups of serous ovarian tumors with representative biological and molecular effects and reclassified them into three major categories: cellular cycle and signaling-related effects, membrane and transport-related effects, and metabolic, immunological, and other effects.

### 3.5. Three Reclassified Categories of the Top 25 Common Dysregulated GO Terms and the Most Relevant Corresponding DEGs

As displayed in Table 3, we reclassified the top 25 most common dysregulated GO terms among serous ovarian tumors into three major categories based on each representative function. There were 9, 8, and 8 GO terms belonging to “cellular cycle and signaling-related effects”, “membrane and transport-related effects”, and “metabolic, immunological, and other effects”, respectively. We sorted the potential genes annotated for all regrouped GO terms among each category with definitions (http://geneontology.org/, accessed on 5 June 2021) and selected the most relevant DEGs with the highest repetitive frequencies determined statistically with cross comparisons. Nine GO terms were reclassified to cellular cycle- and signaling-related effects and four most relevant DEGs were identified with the highest repetition: *EDN1* (endothelin 1), *AKT1* (AKT serine/threonine kinase 1), *IL1B* (interleukin 1 beta), and *INS* (insulin). Eight GO terms were reclassified to membrane- and transport-related effects and the two most relevant DEGs were identified with the highest repetition: *CDK5* (cyclin dependent kinase 5) and *ATP1B1* (sodium/potassium-transporting ATPase subunit beta-1). Eight GO terms were reclassified to metabolic, immunological, and other effects and the seven most relevant DEGs were identified with the highest repetition: *PTK2B* (protein tyrosine kinase 2 beta), *MTOR* (mechanistic target of rapamycin kinase), *APP* (amyloid beta precursor protein), *KIT* (tyrosine-protein kinase KIT), *LEP* (leptin), *MAPK3* (mitogen-activated protein kinase 3), and *SRC* (proto-oncogene tyrosine-protein kinase Src).

### 3.6. The Significant Common Dysfunctional GO-Defined Pathways and Corresponding DEGs

We firstly discovered that there were 5346, 4047, 6779, 7985, and 8251 dysfunctional pathways defined with GO terms in the serous BOT and serous ovarian carcinoma stage I–IV groups, respectively. Then, we placed these pathways in order of correlation for each group according to statistically significant *p*-values. Next, we selected the top 50 most dysfunctional pathways ranked by *p*-value for each disease group to investigate meaningful correlations, as listed in Table 4 and the detailed GO-defined pathways for serous ovarian tumors are listed in Table S5. “Negative regulation of isotype switching (GO:0045829)” ranked first in the serous BOT group; “modified amino acid transmembrane transporter activity (GO:0072349)” ranked first in serous ovarian carcinoma, FIGO stage I; “DNA double-strand break processing involved in repair via single-strand annealing (GO:0010792)” ranked first in serous ovarian carcinoma, FIGO stage II; “DNA double-strand break processing involved in repair via single-strand annealing (GO:0010792)” ranked first in serous ovarian carcinoma, FIGO stage III; and “aryl hydrocarbon receptor binding (GO:0017162)” ranked first in serous ovarian carcinoma, FIGO stage IV. Moreover, we found only one common dysfunctional pathway among the five disease groups of serous ovarian tumors: “aryl hydrocarbon receptor binding (GO:0017162),” which is ranked at 42, 2, 2, 29, and 1 in the groups of serous BOTs and serous ovarian carcinomas of stages I–IV, respectively. Meanwhile, we also found ten corresponding genes, *AHR* (aryl-hydrocarbon receptor), *AIP* (aryl-hydrocarbon receptor-interacting protein), *ARNT* (aryl hydrocarbon receptor nuclear translocator), *ARNT2* (aryl hydrocarbon receptor nuclear translocator 2), ARNTL (aryl hydrocarbon receptor nuclear translocator-like), *NCOA1* (nuclear receptor coactivator 1), *NCOA2* (nuclear receptor coactivator 2), *TAF4* (TATA-box binding protein associated factor 4), *TAF6* (TATA-box binding protein associated factor 6), and *TBP* (TATA box binding protein), with their representative proteins annotated for these GO term-defined dysfunctional pathways acquired from the GO gene set database (http://geneontology.org/, accessed on 5 June 2021).

### 3.7. The Influences of Distinct Valuable DEGs with Corresponding Biomarkers Expressed in Serous Ovarian Tumors

**Figure 3:**
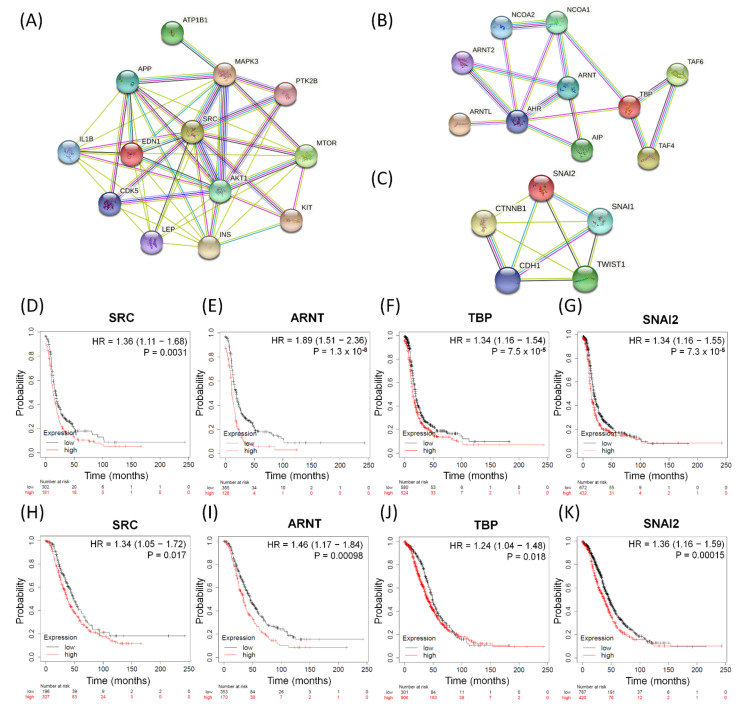
The significant biomarkers influencing serous ovarian tumors. (**A**–**C**) Panels display identified potential involving DEGs subjected to protein–protein interaction (PPI) analysis with interactive network from the STRING database (https://string-db.org (accessed on 21 July 2021)) with intensive interactions. (**A**) All 13 relevant DEGs sorted from the top 25 common dysregulated GO terms, (**B**) all ten DEGs in-volved in dysfunctional aryl hydrocarbon receptor (AHR) binding pathway, and (**C**) five featured DEGs among relevant biomarkers associated with EMT. (**D**–**K**) The four meaningful DEGs (SRC, ARNT, TBP and SNAI2) associated with poor survival outcomes. PFS: (**D**) SRC, (**E**) ARNT, (**F**) TBP, (**G**) SNAI2; OS: (**H**) SRC, (**I**) ARNT, (**J**) TBP, and (**K**) SNAI2 in serous ovarian carcinomas. The hazard ratios of the PFS of SRC, ARNT, TBP, and SNAI2 were 1.36 (1.11–1.68, *p* = 0.0031), 1.89 (1.51–2.36, *p* = 1.3 × 10^−8^), 1.34 (1.16–1.54, *p* = 7.5 × 10^−5^), and 1.34 (1.16–1.55, *p* = 7.3 × 10^−5^), respectively. The hazard ratios of the OS of SRC, ARNT, TBP, and SNAI2 were 1.34 (1.05–1.72, *p* = 0.017), 1.46 (1.17–1.84, *p* = 0.00098), 1.24 (1.04–1.48, *p* = 0.018), 1.36 (1.16–1.59, *p* = 0.00015), respectively.

So far, we have performed GO-based integrative methods to analyze, discover, and reclassify the 25 most important common dysregulated functions among the serous ovarian tumor groups into distinct effective categories and obtained 13 corresponding DEGs in total. We also found one common dysfunctional pathway among the five disease groups and the corresponding ten DEGs. Next, we searched several important biomarkers and relevant genes with close relationships with EMT among ovarian cancers from previous research [50,57,72] and compared them with the DEGs of the top 25 meaningful dysregulated functionomes in this experiment by checking for repetitions and cross comparisons. Five featured DEGs were selected: *CDH1* (cadherin 1), *CTNNB1* (catenin beta 1), *SNAI1* (snail family transcriptional repressor 1, *SNAIL*), *SNAI2* (snail family transcriptional repressor 2, *SLUG*), and *TWIST1* (twist-related protein 1). In addition, we have individually established three functional protein–protein interaction networks using the STRING database (https://string-db.org, accessed on 5 June 2021) based on the relevant DEGs and their corresponding proteins as biomarkers. These networks comprised the following: one, all relevant DEGs sorted from the top 25 common dysregulated functionomes (Figure 3A); two, the 10 DEGs involved in the dysfunctional AHR binding pathway (Figure 3B); and three, the featured DEGs among relevant biomarkers associated with EMT (Figure 3C). All these biomarkers revealed intensive interactions with regulatory cross effects in each network. Simultaneously, we searched the GEO (http://www.ncbi.nlm.nih.gov/geo/, accessed on 5 June 2021) and The Cancer Genome Atlas (TCGA; http://cancergenome.nih.gov, accessed on 5 June 2021) repositories, including datasets downloaded from three major microarray platforms, GPL96 (Affymetrix HG-U133A), GPL570 (Affymetrix HG-U133 Plus 2.0), and GPL571/GPL3921 (Affymetrix HG-U133A 2.0), which contained extracted and corrected raw data of 1232 patients with serous ovarian carcinoma. We entered these datasets with gene expression into the PostgreSQL relational database and compared 28 meaningful DEGs (*EDN1, AKT1, IL1B, INS, CDK5, ATP1B1, PTK2B, MTOR, APP, KIT, LEP, MAPK3, SRC, AHR, AIP, ARNT, ARNT2, ARNTL, NCOA1, NCOA2, TAF4, TAF6, TBP, CDH1, CTNNB1, SNAI1, SNAI2*, and *TWIST1*) selected from the above steps. Then, we calculated and investigated the DEG expression levels, progression-free survival (PFS), and overall survival (OS) among serous ovarian carcinoma patients using the Mann–Whitney test and the receiver operating characteristic test in the R statistical environment (http://www.r-project.org, accessed on 5 June 2021) with Bioconductor libraries (http://www.bioconductor.org, accessed on 5 June 2021) followed by a second normalization to set the average expression of the 22,277 identical probes (http://kmplot.com/analysis/index.php?p=service&cancer=ovar, accessed on 5 June 2021) [73]. Combining all the methods mentioned above, we found that only four DEGs (*SRC, ARNT, TBP*, and *SNAI2*) showed stronger and closer relationships than the other biomarkers in each functional protein–protein interaction network (Figure 3A–C) and had consistent synchronous poor effects on PFS and OS among patients with serous ovarian carcinomas with statistical significance (Figure 3D–K). The high expression levels of the four potentially crucial genes (*SRC, ARNT, TBP*, and *SNAI2*) were significantly correlated with poor prognosis and survival, and the hazard ratios of PFS and OS are shown in each graph below (Figure 3D–K).

### 3.8. Immunohistochemical Validation of Expression Levels for SRC, ARNT, TBP, and SNAI2 among Serous Ovarian Tumors

**Figure 4:**
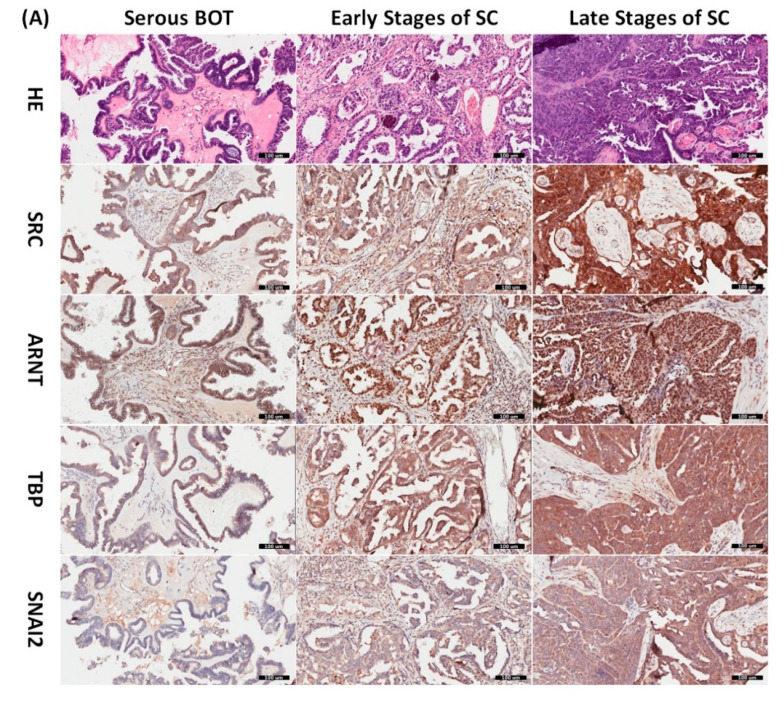

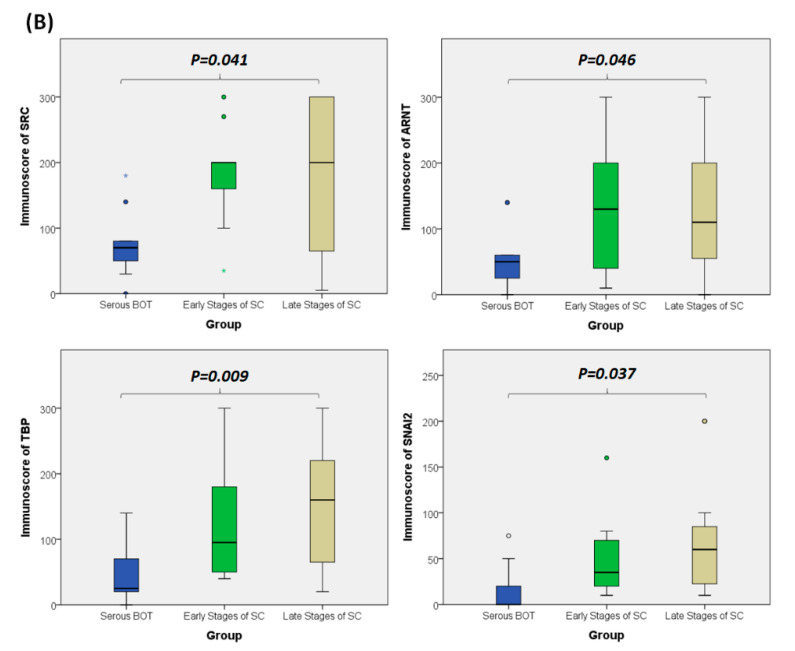
Verified analysis of biomarkers among serous ovarian tumors by IHC staining. (**A**) Clinical samples from patients with serous BOTs (n = 9, left column), early stages of serous ovarian carcinomas (n = 10, middle column), and late stages of serous ovarian carcinomas (n = 31, right column) were immunostained with hematoxylin and eosin (first row), anti-SRC antibody (second row), anti-ARNT antibody (third row), anti-TBP antibody (fourth row), and anti-SNAI2 antibody (fifth row). (**B**) Box plots for expressed biomarkers including SRC, ARNT, TBP, and SNAI2 among groups of serous BOTs (blue), early stages of serous ovarian carcinomas (green), and late stages of serous ovarian carcinomas (light brown). All the expression levels of these meaningful biomarkers were quantified and clearly revealed an increasing trend of mean values from serous BOTs to late stages of serous ovarian carcinomas with statistical significance.

Since the inferred biomarkers including SRC, ARNT, TBP, and SNAI2 from the previous analysis were assumed to be influential in the tumorigenesis of serous ovarian tumors, we gathered relevant clinical samples from a cohort of patients (serous BOT, n = 9; serous ovarian carcinoma, n = 41, including n = 8, 2, 23, and 8 for FIGO stages I–IV, respectively) to explore the clinical characteristics and verify the specific manifestations of the four abovementioned selected DEGs that were determined to participate in the pathogenetic mechanisms of serous ovarian tumors. Because the number of samples in each group was inconsistent, we combined groups as follows to facilitate verification and comparison: serous BOTs, early-stage serous ovarian carcinomas (FIGO stages I and II), and late-stage serous ovarian carcinomas (FIGO stages III and IV). We then performed IHC staining of anti-SRC, anti-ARNT, anti-TBP, and anti-SNAI2 antibodies separately among the three modified disease groups to clinically assess the significant manifestation of SRC, ARNT, TBP, and SNAI2. Professional pathologists verified and interpreted the results evenly and repeatedly throughout the whole diagnostic process using SPSS software (IBM SPSS Statistics version 22.0 for Windows, IBM Corp., Armonk, NY, USA) to quantify the immunoscores of SRC, ARNT, TBP, and SNAI2. The organized results clearly showed that the highest biomarker expression levels tended to occur in the group of late-stage serous ovarian carcinoma, followed by the early-stage group, and lastly the serous BOT group (Figure 4A). We also found that the highest mean values of expression levels for all these biomarkers (SRC, ARNT, TBP, and SNAI2) belonged to the late-stage serous ovarian carcinoma group, with clear increasing trends from the serous BOT group to the late-stage group, and the calculated mean values of the relevant biomarkers were statistically significant (Figure 4B). The detailed results of all scores for relevant featured biomarkers of clinical samples and detailed clinical characteristics of the patients (grade, menopausal status, the presence of BRCA1, BRCA2 mutation, overall survival, and Ca125 level) are listed in Table S6. These results were in accordance with our inferences, implying that many dysregulated functionomes deduced from the integrative GO-based enrichment analysis are dedicated to the pathogenetic mechanisms of serous ovarian tumors. Similarly, these validated results also demonstrated that the dysfunctional AHR binding pathway played a role in the tumorigenesis of serous ovarian tumors. Furthermore, this verification supported the association between EMT and tumor progression. All these significant results confirmed the importance of the previously proposed DEGs and related pathogenic tumorigenesis for serous ovarian tumors.

## 4. Discussion

In this study, we implemented a comprehensive GO-based multi-genome interpretative model using gene set defined functionomes and GSR indices calculated based on gene expression profiles and levels downloaded from public gene set databases to further investigate the complicated and divergent molecular and genetic events of serous ovarian tumors, including serous BOTs and serous ovarian carcinomas at all stages. All results obtained using SVM were statistically significant with high sensitivity, specificity, and accuracy. The GSR indices of all groups of serous ovarian tumors compared to the control groups revealed obvious deviations. The most apparent divergence detected was in the group of serous ovarian carcinoma, FIGO stage IV, and the deviation of serous BOT was just between the early and late stages of serous ovarian carcinomas. Among all groups of serous ovarian tumors, we first identified the top 25 significant common dysregulated functionomes with 13 relevant DEGs, then found one common dysfunctional pathway, AHR binding (GO:0017162), containing 10 corresponding DEGs and excavated five applicable EMT-related DEGs that were related with ovarian neoplasms. Recently, EMT, a reversible process in which epithelial cells acquire mesenchymal cell characteristics due to the loss of cellular polarity and adhesion with increasing cellular migration, has become an important concept in research on tumorigenesis, progression, and chemoresistance of ovarian neoplasms; thus, we included biomarkers of EMT for ovarian tumors in this research. After integrative analysis, including comparison of functional protein–protein interactions and patient survival (PFS and OS) of serous ovarian carcinomas, we obtained four potentially important DEGs: *SRC*, *ARNT*, *TBP*, and *SNAI2*. Finally, IHC validation of these four biomarkers revealed that they significantly increased in samples incrementally from serous BOT to early stages and then to late stages of serous ovarian carcinomas. Since the results obtained in this study are extraordinarily rich and complex, we mainly explained and discussed the crucial dysfunctional AHR binding pathways accompanied by four consequential DEGs that were statistically verified. However, other related meaningful results deserve further exploration and investigation.

Among the preliminary results of GO-based analysis for each group of serous ovarian tumors, we noticed significant differences between serous BOT and serous ovarian carcinomas, that is similar to the divergences of clinical manifestations and histopathological characteristics between the two groups; furthermore, there were also discrepancies even in the four stages of serous ovarian carcinomas. This experiment thus revealed that serous BOTs and serous ovarian carcinomas are basically inconsistent, although all histopathological classifications are confirmed as “serous”. Even so, we identified the top 25 dysregulated functionomes from the first 50 GO-defined terms among the five groups and reclassified them into three categories according to their representative functions. After statistical comparison, we noticed that the category of metabolic and immunological effects had the greatest influence on serous ovarian tumors, followed by membrane and transport-related effects, and lastly, cellular cycle and signaling-related effects. Therefore, we can reasonably infer the importance of the metabolome and immunome in the tumorigenesis of serous ovarian tumors, which require investigation in the future together with the other two effects. In our experiments, we also identified 13 highly relevant DEGs. Many related studies have examined how these DEGs affected the formation of serous ovarian tumors, such as tyrosine kinase related DEGs (*PTK2B*, *KIT*, and *SRC*) [74,75,76], crucial factors known to be related to tumorigenesis (*AKT1*, *MTOR*, *MAPK3*) [64,77,78,79,80], DEGs related to cellular metabolism and immunity (*EDN1*, *IL1B*, *INS*, *APP*, *LEP*) [29,56,60,81,82,83,84,85,86], and agents for signal transmission and channels of cell membranes (*CDK5* and *ATP1B1*) [59,87,88]. Among these DEGs, we found that SRC has consistently poor effects on the survival of serous ovarian carcinoma patients with a poor prognosis of PFS and OS. SRC, a non-receptor protein tyrosine kinase known as a proto-oncogene, participates in the regulation of embryonic development and cell growth [89]. SRC has been found to be activated and overexpressed in association with HER-2/neu overexpression in a high percentage of ovarian cancers, especially in the late stage, and to increase proliferation, angiogenesis, and invasion during tumor development [90]. Silencing of SRC could enhance the cytotoxicity of taxol in ovarian cancer cells to improve the efficacy of chemotherapy [91].

**Figure 5:**
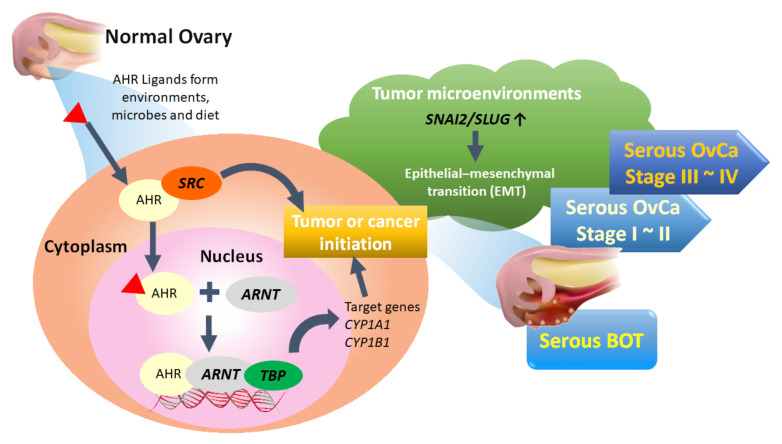
Proposed pathogenetic mechanism of the AHR binding pathway combined with EMT-related factors for tumorigenesis of serous ovarian tumors. BOT: borderline ovarian tumor; OvCa: ovarian carcinoma.

Of the top 50 GO-defined dysfunctional pathways, we found only one meaningful common pathway (AHR binding, GO:0017162) among the five disease groups. We conducted integrated analysis to comprehensively discover the pivotal role of the AHR binding pathway in the tumorigenesis of serous ovarian tumors for the first time. However, in addition to the dysfunctional AHR binding pathway, we also found two common disordered pathways, including positive regulation of keratinocyte differentiation (GO:0045618) and adiponectin secretion (GO:0070162), in all stages of serous ovarian carcinomas. Although not in the top 50 pathways of serous BOTs, these two disordered pathways may be potential problems to be investigated further for the pathogenesis of serous ovarian carcinoma. Through comprehensive analysis, it was revealed that ARNT and TBP have consistently poor effects on PFS and OS. AHR, a ligand-activated transcription factor, is notable for its role in environmental chemical toxicity [92,93,94]; however, in recent studies, AHR was also recognized to play a critical role in tumorigenesis through complex epigenetic and pathogenetic mechanisms encompassing both pro- and anti-tumorigenic activities [95,96]. AHR exists in the cytoplasm and is induced and activated by linking with a group of environmental pollutants as well as other AHR ligands from microbes and diet, and it undergoes certain conformational transformations together with SRC and other cofactors in the cytoplasm to translocate to the nucleus in a dissociated form [95,97,98,99]. AHR can heterodimerize with ARNT, a nuclear translocator, to compose the AhR-ARNT complex, which subsequently binds with specific DNA sequences and xenobiotic response element (XRE) in the enhancer region of certain genes associated with TBP, leading to transcriptional activation of enzymes, such as the cytochrome P450 (CYP) enzymes 1A1 (CYP1A1), CYP1A2, and CYP1B1, for xenobiotic metabolism to induce carcinogenicity of cancer stem cells as tumors or initiate cancer (Figure 5) [100,101,102,103]. Although the current research on the AHR binding pathway and serous ovarian tumors is still limited, it can be roughly understood that the AHR binding pathway influences the formation and occurrence of serous ovarian malignancy through the deep deletion and amplification of AHR transcription factors [95,96,104,105]. Moreover, localization of AHR in the nucleus of tumor cells has been associated with a worse outcome in patients with ovarian cancer, and the role of the AHR/ARNT/CYP-enzyme pathway [106,107] and AHR-driven TBP gene expression in carcinogenesis and cancer initiation, as well as its potential use, have been considered as therapeutic targets for better outcomes [108]. In addition, AHR and NCOA1 discovered in this experiment may also be targets warranting further discussion [98].

Approximately 80% of patients with ovarian cancer suffer from recurrence of metastasis within five years after the initial therapy with debulking operation and chemotherapy due to the development of resistance [109,110]. Accumulating findings have recently demonstrated that EMT may induce chemotherapy resistance and cancer cell stemness by regulating EMT transcription factors, such as Zeb1, Zeb2, Snail, Slug, and Twist1, in a complicated network, and all functional EMT in the tumor microenvironment could exchange tumor cell morphology to upgrade metastatic abilities via migration and invasion [72,111,112,113]. Because avoidance of EMT may be crucial for evaluating and managing tumor metastasis and recurrence [114], we selected five featured DEGs by proofreading and collation with all meaningful DEGs from the top 25 common functionomes of all groups of serous ovarian tumors, and we found that SNAI2 was the most influential DEG due to the concordant results of patient survival. IHC analysis showed an increasing trend from borderline tumors to the late stage of ovarian malignancy. The transcriptional factor SNAI2, also known as SLUG, is considered important for cell migration, differentiation, and metastasis [115,116]. Our study identified the expression and role of SNAI2 in serous ovarian tumors, indicating the progression of serous ovarian tumors possibly through EMT. So far, the association between the AHR binding pathway and EMT among serous ovarian tumors remains unexplored thoroughly, and this experiment provides the opportunity to solve this problem. Aromatic hydrocarbon substances, such as phthalates, di(2-ethylhexyl)phthalate (DEHP), or bisphenol A, are recognized as aggravators, as they upregulate and promote cell proliferation and tumor progression [117,118,119]. In contrast, dietary phytoestrogen and kaempferol could exert anti-carcinogenic and anti-proliferative effects through AHR-related pathways to inhibit the EMT process [120]. Our results showed that there is indeed a tight correlation between AHR and EMT as the degree of malignancy develops in serous ovarian tumors, just like other malignancy [121]. However, how the AHR binding pathway and EMT interact and influence each other in tumor progression and resistance to chemotherapy warrants further research.

This study had several limitations. First, we noticed some limitations in the integrative analytic methods utilized in this study, because the gene set databases of GO terms and related biomolecular pathways did not completely contain or fully define all functionomes of humans. False positivity was attributed to the heterogenicity of disparate cellular histopathological compositions and the indistinguishable elements of different gene sets among the chosen tumor and control samples, and detection by the GSR model was uncertain due to missed errors and untransformed GSR indices if the expression levels were undetectable when converting levels for ordering gene expression. However, these disadvantages may not be obvious in the overall results coupled with the statistically significant high sensitivity, specificity, and accuracy of this experiment. To eliminate these problems in the future, a more precise programming syntax design and more specified sample screening are required. The second limitation is the uneven distribution of case groups. The numbers of serous BOTs, serous ovarian carcinomas, and normal control samples are quite different, and even in the largest population of serous ovarian carcinomas, the numbers of tumors in each stage are quite different. According to the known proportions of serous ovarian tumors, serous ovarian carcinomas account for more EOCs than BOTs, and early stages of serous ovarian carcinomas are usually difficult to diagnose, resulting in fewer diagnoses than at advanced stages. The number of specimens collected for subsequent clinical verification also fits this situation. Although the number of clinical samples is small, with the support of the support vector machine (SVM) used in this study, the preliminary results of this multidisciplinary comprehensive analysis are reliable with high sensitivity, specificity, and accuracy. Even a relatively small number could obtain statistically significant results through IHC verification. Perhaps a more intact gene expression profile database could be constructed to decrease individual discrepancies among ethnic groups in retrospective or prospective cohort studies conducted on a larger scale globally. Third, this study only investigated the common pathogenetic mechanisms of serous ovarian tumors. However, due to the current lack of research, the small number of clinical specimens, and limited funds, data gathered from the GO term database are somewhat obstructed, especially data of serous BOT. Nevertheless, the results are clear and statistically significant, as determined by clinical verification with the immunostaining method. In the future, it may be necessary to gather more specimens, examine more global academic research, and utilize databases of various subtypes to compare and investigate more profoundly and comprehensively the pathogenetic mechanisms with the aid of large-scale experimental tests and funding.

In summary, to investigate potential crucial pathogenetic mechanisms, we performed an integrated GO-based analysis to obtain global genome-wide expression profiles individually and explore meaningful dysregulated functionomes, dysfunctional pathways, and relevant biomarkers of EMT among assorted groups of serous ovarian tumors with the support of elementary machine learning. Based on the above conclusions, we proposed the inferred hypothesis for the formative process of serous ovarian tumor that activated AHR could cooperate with SRC in the cytoplasm to enter cell nuclei and then bind to ARNT together with TBP to act on DNA for initiating targeted AHR-responsive genes to cause tumor or cancer initiation. Besides, biomarker of EMT such as SNAI2 in the tumor microenvironment could also facilitate EMT process accompanied with tumorigenesis (Figure 5). These results provided new directions for understanding the tumorigenesis of serous ovarian tumors and more potential crucial targets for the identification, treatment, monitoring, and even prevention of recurrence combined with targeted therapies as precision medicine in the future.

## 5. Conclusions

Serous ovarian tumors, consisting mainly of serous ovarian carcinoma and serous BOT, are epithelial tumors of the ovary with distinctive characteristics for each subtype. In this study, we made use of integrative analytic methods to select the top 25 significant common GO terms as dysregulated functionomes reclassified into three crucial categories (metabolic, immunological, and other effects; membrane and transport-related effects; and cellular cycle and signaling-related effects) and acquired 13 corresponding DEGs with high probability through cross comparison. For the first time, the dysfunctional AHR binding pathway accompanied with 10 corresponding DEGs was found significantly to be participated in tumorigenesis of both serous BOT and serous ovarian carcinoma and five vital biomarkers related to EMT were searched and gathered for this analytic study. Finally, four important DEGs (SRC, ARNT, TBP, and SNAI2) were compiled to have distinct effects on the survivals of serous ovarian tumor patients with the help of IHC staining for verification showing elevated expression among all clinical samples with increasing malignancy from serous BOT to early stages and to late stages of serous ovarian carcinomas. All acquired results initially supported the inference that dysregulated functionomes with active DEGs and relevant biomarkers could cooperate with the dysfunctional AHR binding pathway together with increased EMT effects in the tumor microenvironment to synergistically influence tumor initiation. These findings considerably contributed to elucidating the pathogenesis of serous ovarian tumors.

## Figures and Tables

**Table 1:** Number of samples and statistics for the groups of serous BOTs and all FIGO stages of serous ovarian carcinomas.

Groups	Sample	Control	Total	Sample Mean (SD ^1^)	Control Mean (SD ^1^)	*p*-Value
Serous BOT ^2^	79	136	215	0.7036 (0.1772)	0.7732 (0.1646)	<0.05
Serous ovarian carcinoma stage I	34	136	170	0.7298 (0.1672)	0.7715 (0.1551)	<0.05
Serous ovarian carcinoma stage II	39	136	175	0.6976 (0.1838)	0.7713 (0.1552)	<0.05
Serous ovarian carcinoma stage III	696	136	832	0.6355 (0.1940)	0.7705 (0.1606)	<0.05
Serous ovarian carcinoma stage IV	131	136	267	0.6147 (0.1969)	0.7706 (0.1565)	<0.05

^1^ SD, standard deviation; ^2^ BOT, borderline ovarian tumor.

**Table 2:** The 50 most dysregulated GO terms for serous BOT and all stages of serous ovarian carcinoma ranked by cluster weight index (CWI).

Groups	Serous BOT		Serous Ovarian Carcinoma Stage I		Serous Ovarian Carcinoma Stage II		Serous Ovarian Carcinoma Stage III		Serous Ovarian Carcinoma Stage IV	
Ranking	GO ID	GO Term	GO ID	GO Term	GO ID	GO Term	GO ID	GO Term	GO ID	GO Term
1	GO:0002682	Regulation of immune system process	GO:0005215	Transporter activity	GO:0044281	Small molecule metabolic process	GO:0002682	Regulation of immune system process	GO:0044281	Small molecule metabolic process
2	GO:0005215	Transporter activity	GO:0044281	Small molecule metabolic process	GO:0005215	Transporter activity	GO:0044281	Small molecule metabolic process	GO:0002682	Regulation of immune system process
3	GO:0001775	Cell activation	GO:0006811	Ion transport	GO:0002682	Regulation of immune system process	GO:0005215	Transporter activity	GO:0005215	Transporter activity
4	GO:0006811	Ion transport	GO:0006629	Lipid metabolic process	GO:0006811	Ion transport	GO:0001775	Cell activation	GO:0051049	Regulation of transport
5	GO:0044281	Small molecule metabolic process	GO:0051049	Regulation of transport	GO:0006629	Lipid metabolic process	GO:0051049	Regulation of transport	GO:0006811	Ion transport
6	GO:0051049	Regulation of transport	GO:0007267	Cell-cell signaling	GO:0051049	Regulation of transport	GO:0006811	Ion transport	GO:0006629	Lipid metabolic process
7	GO:0002252	Immune effector process	GO:0046649	Lymphocyte activation	GO:0001775	Cell activation	GO:0016070	RNA metabolic process	GO:0001775	Cell activation
8	GO:0002520	Immune system development	GO:0040011	Locomotion	GO:0007267	Cell-cell signaling	GO:0045595	Regulation of cell differentiation	GO:0016070	RNA metabolic process
9	GO:0001816	Cytokine production	GO:0055085	Transmembrane transport	GO:0016070	RNA metabolic process	GO:0006629	Lipid metabolic process	GO:0045595	Regulation of cell differentiation
10	GO:0045595	Regulation of cell differentiation	GO:0042592	Homeostatic process	GO:0045595	Regulation of cell differentiation	GO:0040011	Locomotion	GO:0007267	Cell-cell signaling
11	GO:0031399	Regulation of protein modification process	GO:0045595	Regulation of cell differentiation	GO:0046903	Secretion	GO:0001816	Cytokine production	GO:0022008	Neurogenesis
12	GO:0006629	Lipid metabolic process	GO:0051174	Regulation of phosphorus metabolic process	GO:0040011	Locomotion	GO:0002252	Immune effector process	GO:0046903	Secretion
13	GO:0048585	Negative regulation of response to stimulus	GO:0016491	Oxidoreductase activity	GO:0042592	Homeostatic process	GO:0022008	Neurogenesis	GO:0040011	Locomotion
14	GO:0042592	Homeostatic process	GO:0022008	Neurogenesis	GO:0022008	Neurogenesis	GO:0007267	Cell-cell signaling	GO:0042592	Homeostatic process
15	GO:0022610	Biological adhesion	GO:0098772	Molecular function regulator	GO:0007049	Cell cycle	GO:0046903	Secretion	GO:0019219	Regulation of nucleobase-containing compound metabolic process
16	GO:0055085	Transmembrane transport	GO:0031399	Regulation of protein modification process	GO:0060429	Epithelium development	GO:0070727	Cellular macromolecule localization	GO:0002520	Immune system development
17	GO:0051240	Positive regulation of multicellular organismal process	GO:0048585	Negative regulation of response to stimulus	GO:0048585	Negative regulation of response to stimulus	GO:0002520	Immune system development	GO:0051276	Chromosome organization
18	GO:0006915	Apoptotic process	GO:0050865	Regulation of cell activation	GO:0051174	Regulation of phosphorus metabolic process	GO:0051276	Chromosome organization	GO:0060429	Epithelium development
19	GO:0046903	Secretion	GO:0070727	Cellular macromolecule localization	GO:0033043	Regulation of organelle organization	GO:0042592	Homeostatic process	GO:0007049	Cell cycle
20	GO:0060429	Epithelium development	GO:0010817	Regulation of hormone levels	GO:0019219	Regulation of nucleobase-containing compound metabolic process	GO:0007049	Cell cycle	GO:0001816	Cytokine production
21	GO:0051174	Regulation of phosphorus metabolic process	GO:0023056	Positive regulation of signaling	GO:0055085	Transmembrane transport	GO:0051240	Positive regulation of multicellular organismal process	GO:0048585	Negative regulation of response to stimulus
22	GO:0051276	Chromosome organization	GO:0007049	Cell cycle	GO:0002520	Immune system development	GO:0019219	Regulation of nucleobase-containing compound metabolic process	GO:0002252	Immune effector process
23	GO:0007267	Cell-cell signaling	GO:0002520	Immune system development	GO:0051276	Chromosome organization	GO:0033043	Regulation of organelle organization	GO:0051174	Regulation of phosphorus metabolic process
24	GO:0007049	Cell cycle	GO:0015849	Organic acid transport	GO:0031399	Regulation of protein modification process	GO:0031399	Regulation of protein modification process	GO:0031399	Regulation of protein modification process
25	GO:0070727	Cellular macromolecule localization	GO:0007017	Microtubule-based process	GO:0002252	Immune effector process	GO:0060429	Epithelium development	GO:0033043	Regulation of organelle organization
26	GO:0016070	RNA metabolic process	GO:0000003	Reproduction	GO:0070727	Cellular macromolecule localization	GO:0048585	Negative regulation of response to stimulus	GO:0009719	Response to endogenous stimulus
27	GO:0040011	Locomotion	GO:0060089	Molecular transducer activity	GO:0023056	Positive regulation of signaling	GO:0046907	Intracellular transport	GO:0019637	Organophosphate metabolic process
28	GO:0033043	Regulation of organelle organization	GO:0033043	Regulation of organelle organization	GO:0010817	Regulation of hormone levels	GO:0006915	Apoptotic process	GO:0051240	Positive regulation of multicellular organismal process
29	GO:0046907	Intracellular transport	GO:0019219	Regulation of nucleobase-containing compound metabolic process	GO:0016491	Oxidoreductase activity	GO:0051174	Regulation of phosphorus metabolic process	GO:0070727	Cellular macromolecule localization
30	GO:0023056	Positive regulation of signaling	GO:0051240	Positive regulation of multicellular organismal process	GO:0009719	Response to endogenous stimulus	GO:0023056	Positive regulation of signaling	GO:0000003	Reproduction
31	GO:0019219	Regulation of nucleobase-containing compound metabolic process	GO:0046907	Intracellular transport	GO:0098772	Molecular function regulator	GO:0006259	DNA metabolic process	GO:0006915	Apoptotic process
32	GO:0006468	Protein phosphorylation	GO:0022610	Biological adhesion	GO:0006915	Apoptotic process	GO:0055085	Transmembrane transport	GO:0006259	DNA metabolic process
33	GO:0051241	Negative regulation of multicellular organismal process	GO:0050877	Nervous system process	GO:0007010	Cytoskeleton organization	GO:0000003	Reproduction	GO:0007417	Central nervous system development
34	GO:0006259	DNA metabolic process	GO:0030054	Cell junction	GO:0001816	Cytokine production	GO:0044419	Interspecies interaction between organisms	GO:0055085	Transmembrane transport
35	GO:0005102	Signaling receptor binding	GO:0002683	Negative regulation of immune system process	GO:0051240	Positive regulation of multicellular organismal process	GO:0007417	Central nervous system development	GO:0023056	Positive regulation of signaling
36	GO:0098772	Molecular function regulator	GO:0030030	Cell projection organization	GO:0019637	Organophosphate metabolic process	GO:0030030	Cell projection organization	GO:0051241	Negative regulation of multicellular organismal process
37	GO:0080134	Regulation of response to stress	GO:0042127	Regulation of cell proliferation	GO:0046907	Intracellular transport	GO:0065003	Protein-containing complex assembly	GO:0046907	Intracellular transport
38	GO:0022008	Neurogenesis	GO:0042493	Response to drug	GO:0022610	Biological adhesion	GO:0022610	Biological adhesion	GO:0030030	Cell projection organization
39	GO:0015849	Organic acid transport	GO:0023057	Negative regulation of signaling	GO:0000003	Reproduction	GO:0009607	Response to biotic stimulus	GO:0007010	Cytoskeleton organization
40	GO:0044419	Interspecies interaction between organisms	GO:0071495	Cellular response to endogenous stimulus	GO:0030030	Cell projection organization	GO:0007010	Cytoskeleton organization	GO:0098772	Molecular function regulator
41	GO:0023057	Negative regulation of signaling	GO:0051270	Regulation of cellular component movement	GO:0051241	Negative regulation of multicellular organismal process	GO:0009719	Response to endogenous stimulus	GO:0065003	Protein-containing complex assembly
42	GO:0010941	Regulation of cell death	GO:0051338	Regulation of transferase activity	GO:0007417	Central nervous system development	GO:0080134	Regulation of response to stress	GO:0022610	Biological adhesion
43	GO:0042127	Regulation of cell population proliferation	GO:0022603	Regulation of anatomical structure morphogenesis	GO:0007017	Microtubule-based process	GO:0006468	Protein phosphorylation	GO:0009790	Embryo development
44	GO:0009790	Embryo development	GO:0009057	Macromolecule catabolic process	GO:0014070	Response to organic cyclic compound	GO:0032101	Regulation of response to external stimulus	GO:0006468	Protein phosphorylation
45	GO:0002250	Adaptive immune response	GO:0009790	Embryo development	GO:0023057	Negative regulation of signaling	GO:0098772	Molecular function regulator	GO:0016491	Oxidoreductase activity
46	GO:0019637	Organophosphate metabolic process	GO:0051093	Negative regulation of developmental process	GO:0006259	DNA metabolic process	GO:0018193	Peptidyl-amino acid modification	GO:0018193	Peptidyl-amino acid modification
47	GO:0018193	Peptidyl amino acid modification	GO:0043603	Cellular amide metabolic process	GO:0098796	Membrane protein complex	GO:0006952	Defense response	GO:0010817	Regulation of hormone levels
48	GO:0009628	Response to abiotic stimulus	GO:0030855	Epithelial cell differentiation	GO:0042493	Response to drug	GO:0051241	Negative regulation of multicellular organismal process	GO:0080134	Regulation of response to stress
49	GO:0000003	Reproduction	GO:0010876	Lipid localization	GO:0015849	Organic acid transport	GO:0019637	Organophosphate metabolic process	GO:0014070	Response to organic cyclic compound
50	GO:0009719	Response to endogenous stimulus	GO:0051094	Positive regulation of developmental process	GO:0006468	Protein phosphorylation	GO:0044093	Positive regulation of molecular function	GO:0023057	Negative regulation of signaling

**Table 3:** Categorized lists of the top 25 common dysregulated GO terms among serous ovarian tumors reclassified by biological functions and the most relevant corresponding DEGs in each group.

**Cellular Cycle and Signaling Related Effects**		
**GO ID**	**GO Term**	**Most Relevant DEGs**
GO:0045595	Regulation of cell differentiation	*EDN1, AKT1, IL1B, INS*
GO:0007267	Cell-cell signaling	
GO:0042592	Homeostatic process	
GO:0048585	Negative regulation of response to stimulus	
GO:0007049	Cell cycle	
GO:0033043	Regulation of organelle organization	
GO:0051240	Positive regulation of multicellular organismal process	
GO:0023056	Positive regulation of signaling	
GO:0098772	Molecular function regulator	
**Membrane and Transport Related Effects**		
**GO ID**	**GO Term**	**Most Relevant DEGs**
GO:0005215	Transporter activity	*CDK5*, *ATP1B1*
GO:0006811	Ion transport	
GO:0051049	Regulation of transport	
GO:0040011	Locomotion	
GO:0055085	Transmembrane transport	
GO:0070727	Cellular macromolecule localization	
GO:0046907	Intracellular transport	
GO:0022610	Biological adhesion	
**Metabolic, Immunological, and Other Effects**		
**GO ID**	**GO Term**	**Most Relevant DEGs**
GO:0044281	Small molecule metabolic process	*PTK2B*, *MTOR*, *APP*, *KIT*, *LEP*, *MAPK3*, *SRC*
GO:0006629	Lipid metabolic process	
GO:0031399	Regulation of protein modification process	
GO:0051174	Regulation of phosphorus metabolic process	
GO:0019219	Regulation of nucleobase containing compound metabolic process	
GO:0002520	Immune system development	
GO:0022008	Neurogenesis	
GO:0000003	Reproduction	

**Table 4:** The top 50 most dysfunctional GO-defined pathways among serous BOT and all stages of serous ovarian carcinoma ranked by *p*-values.

Groups	Serous BOT		Serous Ovarian Carcinoma Stage I		Serous Ovarian Carcinoma Stage II		Serous Ovarian Carcinoma Stage III		Serous Ovarian Carcinoma Stage IV	
Ranking	GO ID	GO Term	GO ID	GO Term	GO ID	GO Term	GO ID	GO Term	GO ID	GO Term
1	GO:0045829	Negative regulation of isotype switching	GO:0072349	Modified amino acid transmembrane transporter activity	GO:0010792	DNA double-strand break processing involved in repair via single-strand annealing	GO:2001269	Positive regulation of cysteine-type endopeptidase activity involved in apoptotic signaling pathway	GO:0017162	Aryl hydrocarbon receptor binding
2	GO:0008395	Steroid hydroxylase activity	GO:0017162	Aryl hydrocarbon receptor binding	GO:0017162	Aryl hydrocarbon receptor binding	GO:0042908	Xenobiotic transport	GO:0072349	Modified amino acid transmembrane transporter activity
3	GO:0016578	Histone deubiquitination	GO:0097501	Stress response to metal ion	GO:0045002	Double-strand break repair via single-strand annealing	GO:2001267	Regulation of cysteine-type endopeptidase activity involved in apoptotic signaling pathway	GO:0036507	Protein demannosylation
4	GO:0090482	Vitamin transmembrane transporter activity	GO:0016589	NURF complex	GO:0070162	Adiponectin secretion	GO:0015701	Bicarbonate transport	GO:0045618	Positive regulation of keratinocyte differentiation
5	GO:0033499	Galactose catabolic process via UDP-galactose	GO:0055059	Asymmetric neuroblast division	GO:0046643	Regulation of gamma-delta T cell activation	GO:0046007	Negative regulation of activated T cell proliferation	GO:0015106	Bicarbonate transmembrane transporter activity
6	GO:0005347	ATP transmembrane transporter activity	GO:0004865	Protein serine/threonine phosphatase inhibitor activity	GO:0010957	Negative regulation of vitamin D biosynthetic process	GO:0010957	Negative regulation of vitamin D biosynthetic process	GO:0045002	Double-strand break repair via single-strand annealing
7	GO:0015867	ATP transport	GO:0008628	Hormone-mediated apoptotic signaling pathway	GO:0036507	Protein demannosylation	GO:0072608	Interleukin-10 secretion	GO:0045793	Positive regulation of cell size
8	GO:0006825	Copper ion transport	GO:0036507	Protein demannosylation	GO:0046137	Negative regulation of vitamin metabolic process	GO:0090482	Vitamin transmembrane transporter activity	GO:0004016	Adenylate cyclase activity
9	GO:0046007	Negative regulation of activated T cell proliferation	GO:0015106	Bicarbonate transmembrane transporter activity	GO:1990239	Steroid hormone binding	GO:0072350	Tricarboxylic acid metabolic process	GO:0006171	cAMP biosynthetic process
10	GO:0044743	Protein transmembrane import into intracellular organelle	GO:0046643	Regulation of gamma-delta T cell activation	GO:0071360	Cellular response to exogenous dsRNA	GO:0033617	Mitochondrial respiratory chain complex IV assembly	GO:0006517	Protein deglycosylation
11	GO:0050859	Negative regulation of B cell receptor signaling pathway	GO:0005078	MAP-kinase scaffold activity	GO:0044854	Plasma membrane raft assembly	GO:0016409	Palmitoyltransferase activity	GO:0010957	Negative regulation of vitamin D biosynthetic process
12	GO:0099132	ATP hydrolysis coupled cation transmembrane transport	GO:0045618	Positive regulation of keratinocyte differentiation	GO:0016589	NURF complex	GO:0005451	Monovalent cation:proton antiporter activity	GO:0015701	Bicarbonate transport
13	GO:0000244	Spliceosomal tri-snRNP complex assembly	GO:0045793	Positive regulation of cell size	GO:0097501	Stress response to metal ion	GO:0018345	Protein palmitoylation	GO:0036065	Fucosylation
14	GO:0045623	Negative regulation of T-helper cell differentiation	GO:0015116	Sulfate transmembrane transporter activity	GO:0072349	Modified amino acid transmembrane transporter activity	GO:0016417	S-acyltransferase activity	GO:0072497	Mesenchymal stem cell differentiation
15	GO:0004089	Carbonate dehydratase activity	GO:0008271	Secondary active sulfate transmembrane transporter activity	GO:0015106	Bicarbonate transmembrane transporter activity	GO:0046137	Negative regulation of vitamin metabolic process	GO:0050859	Negative regulation of B cell receptor signaling pathway
16	GO:0033270	Paranode region of axon	GO:0004016	Adenylate cyclase activity	GO:0050428	3′-phosphoadenosine 5′-phosphosulfate biosynthetic process	GO:0002370	Natural killer cell cytokine production	GO:0019870	Potassium channel inhibitor activity
17	GO:0050686	Negative regulation of mRNA processing	GO:0006171	cAMP biosynthetic process	GO:0001765	Membrane raft assembly	GO:0045618	Positive regulation of keratinocyte differentiation	GO:0036066	Protein O-linked fucosylation
18	GO:0044183	Protein binding involved in protein folding	GO:0008272	Sulfate transport	GO:0019531	Oxalate transmembrane transporter activity	GO:0016589	NURF complex	GO:0046137	Negative regulation of vitamin metabolic process
19	GO:0061082	Myeloid leukocyte cytokine production	GO:0022821	Potassium ion antiporter activity	GO:0045618	Positive regulation of keratinocyte differentiation	GO:0015924	Mannosyl-oligosaccharide mannosidase activity	GO:0044322	Endoplasmic reticulum quality control compartment
20	GO:0000002	Mitochondrial genome maintenance	GO:0015924	Mannosyl-oligosaccharide mannosidase activity	GO:0008271	Secondary active sulfate transmembrane transporter activity	GO:0045616	Regulation of keratinocyte differentiation	GO:0004865	Protein serine/threonine phosphatase inhibitor activity
21	GO:0045591	Positive regulation of regulatory T cell differentiation	GO:0019373	Epoxygenase P450 pathway	GO:0015116	Sulfate transmembrane transporter activity	GO:0070162	Adiponectin secretion	GO:0007175	Negative regulation of epidermal growth factor-activated receptor activity
22	GO:2001182	Regulation of interleukin-12 secretion	GO:0019532	Oxalate transport	GO:0032184	SUMO polymer binding	GO:0022821	Potassium ion antiporter activity	GO:0042359	Vitamin D metabolic process
23	GO:0097503	Sialylation	GO:0099509	Regulation of presynaptic cytosolic calcium ion concentration	GO:0045586	Regulation of gamma-delta T cell differentiation	GO:0015377	Cation: chloride symporter activity	GO:0009975	Cyclase activity
24	GO:0008373	Sialyltransferase activity	GO:0019531	Oxalate transmembrane transporter activity	GO:0019532	Oxalate transport	GO:0015379	Potassium: chloride symporter activity	GO:0045616	Regulation of keratinocyte differentiation
25	GO:0008385	Ikappab kinase complex	GO:0050428	3′-phosphoadenosine 5′-phosphosulfate biosynthetic process	GO:0071447	Cellular response to hydroperoxide	GO:0098719	Sodium ion import across plasma membrane	GO:0016849	Phosphorus-oxygen lyase activity
26	GO:0072643	Interferon-gamma secretion	GO:0008391	Arachidonic acid monooxygenase activity	GO:0042363	Fat-soluble vitamin catabolic process	GO:0072643	Interferon-gamma secretion	GO:1990239	Steroid hormone binding
27	GO:0031248	Protein acetyltransferase complex	GO:0097267	Omega-hydroxylase P450 pathway	GO:0072497	Mesenchymal stem cell differentiation	GO:0097503	Sialylation	GO:0071305	Cellular response to vitamin D
28	GO:0000188	Inactivation of MAPK activity	GO:0010792	DNA double-strand break processing involved in repair via single-strand annealing	GO:0005337	Nucleoside transmembrane transporter activity	GO:0008373	Sialyltransferase activity	GO:0070162	Adiponectin secretion
29	GO:0002634	Regulation of germinal center formation	GO:0031010	ISWI-type complex	GO:0008272	Sulfate transport	GO:0017162	Aryl hydrocarbon receptor binding	GO:0006895	Golgi to endosome transport
30	GO:2000515	Negative regulation of CD4-positive, alpha-beta T cell activation	GO:0071360	Cellular response to exogenous dsRNA	GO:0002175	Protein localization to paranode region of axon	GO:0032689	Negative regulation of interferon-gamma production	GO:0097225	Sperm midpiece
31	GO:2000320	Negative regulation of T-helper 17 cell differentiation	GO:0006685	Sphingomyelin catabolic process	GO:0019373	Epoxygenase P450 pathway	GO:0015296	Anion:cation symporter activity	GO:2000535	Regulation of entry of bacterium into host cell
32	GO:0060907	Positive regulation of macrophage cytokine production	GO:0071577	Zinc ion transmembrane transport	GO:2001182	Regulation of interleukin-12 secretion	GO:0046643	Regulation of gamma-delta T cell activation	GO:0004198	Calcium-dependent cysteine-type endopeptidase activity
33	GO:0032426	Stereocilium tip	GO:0005385	Zinc ion transmembrane transporter activity	GO:0090177	Establishment of planar polarity involved in neural tube closure	GO:0042788	Polysomal ribosome	GO:0019373	Epoxygenase P450 pathway
34	GO:0046915	Transition metal ion transmembrane transporter activity	GO:0044854	Plasma membrane raft assembly	GO:0015858	Nucleoside transport	GO:0045503	Dynein light chain binding	GO:1905276	Regulation of epithelial tube formation
35	GO:0033549	MAP kinase phosphatase activity	GO:0099516	Ion antiporter activity	GO:0031095	Platelet dense tubular network membrane	GO:2000773	Negative regulation of cellular senescence	GO:0071313	Cellular response to caffeine
36	GO:0016854	Racemase and epimerase activity	GO:0038044	Transforming growth factor-beta secretion	GO:0019870	Potassium channel inhibitor activity	GO:0002707	Negative regulation of lymphocyte mediated immunity	GO:0045671	Negative regulation of osteoclast differentiation
37	GO:0043371	Negative regulation of CD4-positive, alpha-beta T cell differentiation	GO:0097264	Self-proteolysis	GO:0034139	Regulation of toll-like receptor 3 signaling pathway	GO:0070234	Positive regulation of T cell apoptotic process	GO:0099516	Ion antiporter activity
38	GO:0030890	Positive regulation of B cell proliferation	GO:0045852	pH elevation	GO:0101020	Estrogen 16-alpha-hydroxylase activity	GO:0071447	Cellular response to hydroperoxide	GO:0019532	Oxalate transport
39	GO:0050798	Activated T cell proliferation	GO:0009698	Phenylpropanoid metabolic process	GO:0002933	Lipid hydroxylation	GO:0099587	Inorganic ion import across plasma membrane	GO:2001222	Regulation of neuron migration
40	GO:0015662	ATPase activity, coupled to transmembrane movement of ions, phosphorylative mechanism	GO:2001182	Regulation of interleukin-12 secretion	GO:0060353	Regulation of cell adhesion molecule production	GO:0071636	Positive regulation of transforming growth factor beta production	GO:0016712	Oxidoreductase activity, acting on paired donors, with incorporation or reduction of molecular oxygen, reduced flavin or flavoprotein as one donor, and incorporation of one atom of oxygen
41	GO:0048291	Isotype switching to IgG isotypes	GO:0015491	Cation: cation antiporter activity	GO:0008391	Arachidonic acid monooxygenase activity	GO:0033962	Cytoplasmic mRNA processing body assembly	GO:2000833	Positive regulation of steroid hormone secretion
42	GO:0017162	Aryl hydrocarbon receptor binding	GO:0071313	Cellular response to caffeine	GO:1905276	Regulation of epithelial tube formation	GO:0043189	H4/H2A histone acetyltransferase complex	GO:0007077	Mitotic nuclear envelope disassembly
43	GO:0061081	Positive regulation of myeloid leukocyte cytokine production involved in immune response	GO:2000833	Positive regulation of steroid hormone secretion	GO:0031010	ISWI-type complex	GO:0043968	Histone H2A acetylation	GO:0015924	Mannosyl-oligosaccharide mannosidase activity
44	GO:0016226	Iron-sulfur cluster assembly	GO:0070162	Adiponectin secretion	GO:0045671	Negative regulation of osteoclast differentiation	GO:2001222	Regulation of neuron migration	GO:2001182	Regulation of interleukin-12 secretion
45	GO:0030007	Cellular potassium ion homeostasis	GO:0031995	Insulin-like growth factor II binding	GO:0031995	Insulin-like growth factor II binding	GO:0070670	Response to interleukin-4	GO:0015373	Anion: sodium symporter activity
46	GO:0015701	Bicarbonate transport	GO:0005451	Monovalent cation: proton antiporter activity	GO:0008541	Proteasome regulatory particle, lid subcomplex	GO:0018230	Peptidyl-L-cysteine S-palmitoylation	GO:0090177	Establishment of planar polarity involved in neural tube closure
47	GO:0071850	Mitotic cell cycle arrest	GO:0008541	Proteasome regulatory particle, lid subcomplex	GO:0052173	Response to defenses of other organism involved in symbiotic interaction	GO:0005416	Amino acid: cation symporter activity	GO:0016725	Oxidoreductase activity, acting on CH or CH2 groups
48	GO:0071014	Post-mRNA release spliceosomal complex	GO:0031095	Platelet dense tubular network membrane	GO:0038085	Vascular endothelial growth factor binding	GO:0006825	Copper ion transport	GO:0002335	Mature B cell differentiation
49	GO:0008242	Omega peptidase activity	GO:0016712	Oxidoreductase activity, acting on paired donors, with incorporation or reduction of molecular oxygen, reduced flavin or flavoprotein as one donor, and incorporation of one atom of oxygen	GO:0005310	Dicarboxylic acid transmembrane transporter activity	GO:0002857	Positive regulation of natural killer cell-mediated immune response to tumor cell	GO:0009111	Vitamin catabolic process
50	GO:0043966	histone H3 acetylation	GO:0050859	Negative regulation of B cell receptor signaling pathway	GO:2000270	Negative regulation of fibroblast apoptotic process	GO:0000185	Activation of MAPKKK activity	GO:0019843	rRNA binding

## Data Availability

The gene set databases of microarrays expression profiles are publicly available and downloaded from the National Center for Biotechnology Information (NCBI) Gene Expression Omnibus (GEO) repository (https://www.ncbi.nlm.nih.gov/geo/, accessed on 5 June 2021).

## Supplementary Materials

The following are available online at https://www.mdpi.com/article/10.3390/biomedicines9080866/s1, Table S1, Detailed informative data of all collected samples. Table S2, Sensitivity, specificity, and accuracy of binary classification and prediction by supervised machine learning. Table S3, The whole deregulated GO terms of serous ovarian tumors. Table S4. The top 25 common dysregulated GO terms among the five case groups (serous BOT and all stages of serous ovarian carcinoma). Table S5, The whole dysfunctional GO pathways for serous ovarian tumors. Table S6, All IHC scores for relevant biomarkers of clinical samples and detailed clinical characteristics of the patients.
