# Supplementary material for: Synergistic AHR Binding Pathway with EMT Effects on Serous Ovarian Tumors Recognized by Multidisciplinary Integrated Analysis
Source: Biomedicines. 2021 Jul 22;9(8):866. doi: 10.3390/biomedicines9080866 (PMC8389648; doi:10.3390/biomedicines9080866)
Supplement: Supplementary file 1 [file biomedicines-09-00866-s001.zip › Table S4 The top 25 common dysregulated GO terms among the five case groups (serous BOT and all stages of serous ovarian carcinoma).pdf]

Table S4. The top 25 common dysregulated GO terms among the five case groups (serous BOT and all stages of serous ovarian carcinoma)

Order	GO ID	GO term	Original ranking in serous BOT	Original ranking in serous ovarian carcinoma, FIGO stage I	Original ranking in serous ovarian carcinoma, FIGO stage II	Original ranking in serous ovarian carcinoma, FIGO stage III	Original ranking in serous ovarian carcinoma, FIGO stage IV
1	GO:0044281	Small molecule metabolic process	5	2	1	2	1
2	GO:0005215	Transporter activity	2	1	2	3	3
3	GO:0006811	Ion transport	4	3	4	6	5
4	GO:0051049	Regulation of transport	6	5	6	5	4
5	GO:0006629	Lipid metabolic process	12	4	5	9	6
6	GO:0045595	Regulation of cell differentiation	8	9	10	8	9
7	GO:0007267	Cell-cell signaling	23	6	8	14	10
8	GO:0040011	Locomotion	27	8	12	10	13
9	GO:0042592	Homeostatic process	14	10	13	19	14
10	GO:0002520	Immune system development	8	23	22	17	16
11	GO:0022008	Neurogenesis	38	14	14	13	11
12	GO:0048585	Negative regulation of response to stimulus	13	17	17	26	21
13	GO:0031399	Regulation of protein modification process	11	16	24	24	24
14	GO:0007049	Cell cycle	24	22	15	20	19
15	GO:0051174	Regulation of phosphorus metabolic process	21	12	18	29	23
16	GO:0055085	Transmembrane transport	16	10	21	32	34

17	GO:0070727	Cellular macromolecule localization	25	19	26	16	29
18	GO:0019219	Regulation of nucleobase-containing compound metabolic process	31	29	20	22	15
19	GO:0033043	Regulation of organelle organization	28	28	19	23	25
20	GO:0051240	Positive regulation of multicellular organismal process	17	30	35	21	28
21	GO:0023056	Positive regulation of signaling	30	21	27	30	35
22	GO:0046907	Intracellular transport	29	31	37	27	37
23	GO:0022610	Biological adhesion	15	32	38	38	42
24	GO:0098772	Molecular function regulator	36	15	31	45	40
25	GO:0000003	Reproduction	49	26	39	33	30
