# Supplementary material for: Synergistic AHR Binding Pathway with EMT Effects on Serous Ovarian Tumors Recognized by Multidisciplinary Integrated Analysis
Source: Biomedicines. 2021 Jul 22;9(8):866. doi: 10.3390/biomedicines9080866 (PMC8389648; doi:10.3390/biomedicines9080866)
Supplement: Supplementary file 1 [file biomedicines-09-00866-s001.zip › Table S2 Sensitivity, specificity, and accuracy of binary classification and prediction by supervised machine learning.pdf]

Table S2. Sensitivity, specificity, and accuracy of binary classification and prediction by supervised machine learning

Gene set	Classification	Groups	Sensitivity (SD[1])	Specificity (SD[1])	Accuracy (SD[1])	AUC[2]
Gene Ontology resources	Binary	Serous BOT[3]	0.9933 (0.0211)	1.0000 (0.0000)	0.9977 (0.0073)	0.9967
		Serous ovarian carcinoma stage I	0.9575 (0.0567)	1.0000 (0.0000)	0.9930 (0.0091)	0.9794
		Serous ovarian carcinoma stage II	0.9513 (0.0587)	1.0000 (0.0000)	0.9897 (0.0121)	0.9771
		Serous ovarian carcinoma stage III	1.0000 (0.0000)	0.9985 (0.0049)	0.9998 (0.0008)	0.9993
		Serous ovarian carcinoma stage IV	1.0000 (0.0000)	1.0000 (0.0000)	1.0000 (0.0000)	1.0000

[1] SD, standard deviation; [2] AUC, area under the curve; [3] BOT, borderline ovarian tumor.
